# Systems Level Modeling of the Cell Cycle Using Budding Yeast

**Published:** 2007-12-11

**Authors:** B.P. Ingalls, B.P. Duncker, D.R. Kim, B.J. McConkey

**Affiliations:** 1 Department of Applied Mathematics, University of Waterloo; 2 Department of Biology, University of Waterloo

**Keywords:** cell cycle, budding yeast, proteomics, dynamic modeling

## Abstract

Proteins involved in the regulation of the cell cycle are highly conserved across all eukaryotes, and so a relatively simple eukaryote such as yeast can provide insight into a variety of cell cycle perturbations including those that occur in human cancer. To date, the budding yeast *Saccharomyces cerevisiae* has provided the largest amount of experimental and modeling data on the progression of the cell cycle, making it a logical choice for in-depth studies of this process. Moreover, the advent of methods for collection of high-throughput genome, transcriptome, and proteome data has provided a means to collect and precisely quantify simultaneous cell cycle gene transcript and protein levels, permitting modeling of the cell cycle on the systems level. With the appropriate mathematical framework and sufficient and accurate data on cell cycle components, it should be possible to create a model of the cell cycle that not only effectively describes its operation, but can also predict responses to perturbations such as variation in protein levels and responses to external stimuli including targeted inhibition by drugs. In this review, we summarize existing data on the yeast cell cycle, proteomics technologies for quantifying cell cycle proteins, and the mathematical frameworks that can integrate this data into representative and effective models. Systems level modeling of the cell cycle will require the integration of high-quality data with the appropriate mathematical framework, which can currently be attained through the combination of dynamic modeling based on proteomics data and using yeast as a model organism.

## Introduction

The development and spread of cancer involves the interplay of a vast number of factors involved in processes such as cell cycle progression, preservation of genome integrity, apoptosis, angiogenesis and metastasis, among others. The way in which these factors interact with one another can vary greatly between different forms of cancer and thus diagnostic and prognostic indicators for a particular cancer type are often poor indicators for cancers affecting cells in other organs or tissues. One of the best hopes for grappling with such complexity is the use of systems biology.

A thorough understanding of cell cycle regulation is of central importance to obtaining an accurate description of cancer development. A hallmark of cancer is the breakdown of the normal mechanisms governing cell proliferation and so determining precisely how these processes work is key to the development of improved cancer diagnostics and therapeutics. In addressing the cell division cycle, the foundations for these efforts have already been laid in a series of mathematical representations of cell cycle progression in the budding yeast *Saccharomyces cerevisiae.*

Effective systems level modeling will require high quality data sets. Recent advances in proteomics methods promise to deliver high quality qualitative and quantitative data which, when combined with dynamic mathematical approaches, will provide more accurate and predictive models of the genesis and spread of cancer than are currently available. In this review, we describe the characteristics of *S. cerevisiae* that make this organism particularly useful for systems biology approaches, survey proteomics tools for collecting systems level data, and assess the progress that has already been made in modeling the eukaryotic cell cycle.

## The Use of *Saccharomyces cerevisiae* in Systems Biology

The budding yeast *Saccharomyces cerevisiae* has long been a leading model organism for cell cycle studies. The numerous advantages to working with *S. cerevisiae* include the fact that it is easily grown in the lab, has a comparatively short generation time (typically 90 minutes for a wild type strain at 30 °C) and has less susceptibility to contamination relative to most other eukaryotic cell cultures. Many mutant strains for genes encoding factors involved in a wide array of cellular functions have been identified in budding yeast. A large proportion of these are temperature-sensitive conditional mutants, for which the protein product of the mutant allele is functional at one (permissive) temperature and inactive at another (restrictive) temperature. These strains are ideally suited for simple temperature-shift experiments to study a protein’s function and role within a network. Homologous recombination can be efficiently used in *S. cerevisiae* to manipulate the genome more easily than is currently possible for most other model organisms, which allows researchers to delete, mutate and epitope-tag specific genes, as well as replace their upstream regulatory elements (Amberg et al. 2005).

In somatic cells, the process of cell division can be divided into four stages: G_1_ phase, during which cells grow and monitor the external environment to decide whether to exit the cell cycle (G_0_) or commit to cell division; S phase, during which the genome is duplicated; G_2_ phase, during which the cells prepare for mitosis; and finally M phase, during which the chromosomes are partitioned and cytokinesis occurs resulting in two cells. Most mutations resulting in human cancer are in genes encoding factors involved in the transition of cells from G_1_ phase to S phase (reviewed in Sidorova and Breeden, 2003). Many of these factors were originally isolated and characterized in budding yeast, and their human orthologues have recently shown great promise as biomarkers for early cancer detection (reviewed in Semple and Duncker, 2004). The use of systems biology to develop models of the yeast cell cycle, and the G_1_ to S transition in particular, holds out great hope not only for the development of new diagnostic biomarkers, but for identifying promising new drug targets for cancer therapy.

The complete genome sequence of *S. cerevisiae* was published in 1996 (Goffeau et al. 1996), representing the first time this had been accomplished for a eukaryote. Combined with the aforementioned genetic tractability, this knowledge opened the floodgates for a large number of ambitious studies of the budding yeast genome and proteome. These include genome level analyses of expression via microarrays and proteomics techniques, functional perturbation via deletion and overexpression studies, and localization and interaction studies via tagged mutant strains, as discussed below.

## High-Throughput Functional Analyses

Of all the aspects of cell biology that have been studied in budding yeast, none have been more thoroughly investigated than the mechanisms governing cell cycle progression. Stemming from the pioneering genetic screens of Leland Hartwell (Hartwell et al. 1970; Culotti and Hartwell, 1971; Hartwell, 1971a; Hartwell, 1971b), a large number of temperature-sensitive cdc (cell division cycle) mutants were identified for genes important for such processes as DNA replication, nuclear division and cytokinesis. More recently, genomic level functional characterization has been investigated through systematic gene deletion (Winzeler et al. 1999) protein over-expression (Sopko et al. 2006), and protein localization through GFP fusions (Huh et al. 2003). Composition of large complexes can now also be effectively investigated by mass spectrometry, for example as demonstrated by the identification of proteins within yeast nuclear pore complexes (Rout et al. 2000) and within spindle pole bodies (Wigge et al. 1998). A number of high throughput procedures have benefited from automation of various steps, an approach to which budding yeast is exceptionally well suited. For example, Boone and colleagues have developed an ordered array of the ~4700 viable yeast gene-deletion mutant strains which can be mated with specific mutant query strains to identify synthetic lethal interactions using automated robotic pinning (Tong et al. 2001). Synthetic lethality or synthetic sickness can arise when mutant alleles for redundant pathways, which individually have no phenotypic consequences, are combined through mating of individual haploid yeast strains followed by sporulation to produce new haploids. This has proven to be a powerful approach to identify epistatic relationships between genes (Jorgensen et al. 2002, Goehring et al. 2003, Suter et al. 2004, Audhya et al. 2004, Wong et al. 2004, Chang et al. 2005, Davierwala et al. 2005, Measday et al. 2005).

An exciting recent use of the deletion mutant strain collection is chemical-genetic profiling, in which hypersensitivity to 82 compounds and natural extracts was assessed (Parsons et al. 2006). Compounds with similar patterns of effects on the deletion mutants were clustered together giving insight as to which cellular pathways they might be affecting, providing a powerful new means for assessing the potential of novel pharmaceutical and natural treatments. Drug sensitivities have also been tested in haploids with increased dosage of specific genes and with a set of heterozygous diploid strains representing deletions in ~5000 nonessential and ~1000 essential genes (reviewed in Sturgeon et al. 2006).

The interactions of proteins provide another key component that can be used for elucidating function. Two high-throughput methods for identifying interactions between proteins are yeast two-hybrid (Uetz et al. 2000, Ito et al. 2001) and affinity purification (Gavin et al. 2006, Ho et al. 2002, Krogan et al. 2006), which identify physical pairwise protein-protein interactions and members of protein complexes respectively. Yeast two hybrid methods identify interactions via binding between two recombinant fusion proteins, one known as the “bait” which includes a DNA binding domain that targets it upstream of a reporter gene, and another termed the “prey” which includes a transcriptional activation domain (reviewed in Parrish et al. 2006). The extent to which the proteins fused to these two domains interact can be determined from the expression levels of the reporter gene. While this method is susceptible to false positives and false negatives, it has provided the bulk of binary interaction data currently available (Parrish et al. 2006).

Affinity purification methods usually utilize an epitope-tagged target protein to selectively purify proteins binding with the target protein of interest. Although a variety of methods exist, high-throughput analyses have typically used either tandem affinity purification (TAP) or flag-tagging (reviewed in Gingras et al. 2007). Tandem affinity purification (TAP) uses proteins tagged with two epitopes, and can selectively extract protein complexes from whole cell extracts through sequential affinity columns corresponding to each of the epitopes. Flag-tagging adopts a similar approach, using a one-step purification via flag-tag specific antibodies immobilized on a resin column. In each case, proteins interacting with the tagged protein will be co-purified, and can be identified by mass spectrometry. These techniques have been used effectively to survey complexes present within the yeast proteome (Krogan et al. 2006; Gavin et al. 2006; Ho et al. 2002). The data generated by these high throughput studies typically requires processing to separate the true interaction complexes from false positives, using methods such as reciprocal interactions and clustering algorithms (discussed in Gingras, 2007). It is possible that some transient interactions may be identified using similar procedures, though to date they have been primarily applied to the identification of protein complexes.

A promising technique for identification of protein complexes and potential identification of transient interactions is fluorescence resonance energy transfer (FRET). FRET has been used to elucidate the architecture of the spindle pole body (Muller et al. 2005) by developing a set of protein-protein distance constraints based on signal intensities. In theory, this method could be effectively applied to identify transient protein-protein interactions within a functioning cell.

Studies such as these have provided a wealth of new resources now available for yeast research. These include strain collections for systematic gene deletion (Winzeler et al. 1999, available from EUROSCARF, http://web.uni-frankfurt.de/fb15/mikro/euroscarf/), TAP (tandem affinity purification)-fusions (Ghaemmaghami et al. 2003, available from Open Biosystems; Gavin et al. 2006, available from EUROSCARF), GFP fusions (Huh et al. 2003, available from Invitrogen), and titratable Tet-promoters (Mnaimneh et al. 2004, available from Open Biosystems). Databases with the results of many of the high-throughput screens are also available, as well as a variety of tools for visualizing and managing systems-level data (Table 1).

## Global Expression Analysis of the Cell Cycle

Genomics and proteomics tools can be used to probe dynamic function by monitoring changes in the abundance of the network components. These data could include large scale monitoring of gene expression via microarrays or monitoring of protein expression via proteomics techniques. The first genome-wide studies of gene expression in yeast (Cho et al. 1998; Spellman et al. 1998) used microarrays to successfully identify over 800 cyclically expressed genes correlated to the cell cycle. More recently, Pramila et al. (2006) obtained comparable data and further identified promoter elements and transcription factors specific to individual phases of the yeast cell cycle. Similarly, 750 cyclically regulated genes were also identified in the cell cycle of the fission yeast *Schizosaccharomyces pombe* (Oliva et al. 2005), with a considerable overlap in the genes, transcription factors, and regulatory mechanisms between the two species in spite of over a billion years since divergence. The consistency across widely divergent species permits ready comparison of cell cycle genes and proteins across eukaryotic organisms, providing further utility for yeast as a model organism for investigating the cell cycle.

Systems level investigation of gene expression provides considerable information on the function and regulation of genes and protein products. However, as proteins are the predominant functional units within a network and gene expression is only moderately correlated with protein expression, gene expression provides an incomplete picture from a modeling perspective. Proteomic quantitation is thus a valuable complement to genetic data and development of quantitative proteomics methods is key in a systems biology context. Proteomics technologies provide a means to separate, identify, and potentially quantify the set of protein isoforms expressed within a cell or tissue. Two methods have become widely used in proteomics studies: 2D gel electrophoresis (a ‘top down’ approach, separating intact proteins prior to identification), and liquid chromatography (a ‘bottom up’ approach, separating peptide fragments from complex protein digests).

2D gel methods typically use a first dimension separation by charge, in which components within a complex protein mixture are separated by isoelectric point. Separation in the second dimension is by molecular weight, using sodium dodecyl sulfate polyacrylamide gel electrophoresis (SDSPAGE). Separated proteins are visualized by silver stain, Coomassie blue dyes, or fluorescent dyes. Depending on the system used, it is possible to visualize hundreds to more than a thousand unique proteins. Once separated, proteins of interest are excised from the gel matrix, digested, and identified by mass spectrometry. 2D gel methods have been applied to *S. cerevisiae*, resulting in a set of reference maps for the yeast proteome (for example, Perrot et al. 1999; Wildgruber et al. 2002). While these maps are useful guides, subsequent 2D gel experiments typically require confirmation of protein IDs by mass spectrometry. With respect to systems-level modeling, an advantage of gelbased methodologies is that post-translational modifications and different isoforms of the same protein can be effectively monitored.

The establishment of proteome maps provides a list of proteins present under given experimental conditions. However, by itself this provides little information on the roles of specific proteins. To identify changes in abundance, two or more experimental conditions are compared to identify differentially regulated proteins, similar to what is accomplished at the transcript level in microarray analysis. There has been a variety of comparative expression studies in yeast, such as investigations of differences in protein expression under glucose limitation or ethanol limitation (Kolkman et al. 2005), cadmium exposure (Vido et al. 2001) or amino acid starvation (Yin et al. 2004). Similar methodologies can be used with synchronized yeast cultures to obtain cell cycle data, though detection of low-abundance proteins remains challenging.

Issues with reproducibility in earlier 2D gel-based methods have led to the development of techniques such as Differential In Gel Electrophoresis (DIGE, Tonge et al. 2001), in which two samples plus an internal standard are labeled with different fluorescent dyes, which are then combined and run on a standard 2D gel. Proteins from the different samples co-migrate to the same spot on the 2D gel, and DIGE can thus identify proteins up- or down-regulated under the given experimental conditions. Experiments can be designed such that the samples represent multiple points within a dynamic time-series, providing coarse-grained information on dynamic responses. Quantitative 2D gel based methods such as these can thus provide extensive information on differentially expressed proteins and post-translational modifications via different protein isoforms.

The other primary means of collecting proteomic data is liquid chromatography/mass spectrometry (LC/MS), which replaces the gel-based charge and molecular weight separation of proteins with liquid phase separation of peptides. Separation in two dimensions is often used here as well, such as ion exchange followed by separation according to hydrophobicity. One example of a versatile LC method is multi-dimensional protein identification technology, or MudPIT (Link et al. 1999; Wolters et al. 2001). In this technique, a sample such as a whole cell extract is digested and separated by 2D-LC directly coupled to a tandem mass spectrometer. This technique has been successfully applied to the yeast genome (Washburn et al. 2001; Graumann et al. 2004). Recently this method was expanded by including a third dimension of separation, resulting in identification of 3109 yeast proteins (Wei et al. 2005). A potential drawback of LC methods is the variability of MS peak intensities, making direct quantification difficult. However, several strategies have been developed to quantify differences in proteins using LC-MS based methods, which can be grouped depending on labeling procedures: metabolic labeling, chemical labeling of thiol groups, enzymatic isotopic labeling, derivatization of the N- or C-terminus of peptides, lysine specific labeling, phosphopeptide labeling, and differential mass mapping (for a general review and comparison see Lill, 2003). Isotopes may be incorporated at the metabolic level by including stable isotopes as a component within a food source, at the protein level, or at the peptide level (reviewed in Julka and Regnier, 2004; Sechi and Oda, 2003). Among these methods, various applications of Isotope Coded Affinity Tags (ICAT) and Stable Isotope Labeling with Amino acids in Cell culture (SILAC) have been utilized in proteomic analyses (reviewed in Gingras et al. 2005). In principle, these methods exploit small mass differences between heavy and light isotopes of chemically indistinguishable peptides. SILAC involves metabolic incorporation of heavy isotopes such as ^13^C using labeled amino acids (Ong et al. 2002). In contrast to *in vivo* metabolic labeling, chemical attachment after protein extraction provides another strategy for comparing protein samples. This requires modification of protein and peptides with reactive chemicals containing isotopic mass differences and can be accomplished using ICAT methods (Gygi et al. 1999), in which cysteines are labeled with either a light or heavy isotopic tag. A similar labeling can be done at the peptide level by digestion in ^18^O labeled water or by derivatization by methods such as iTRAQ (Ross et al. 2004). The iTRAQ methodology is particularly appealing for complex samples, as different isoforms of a given differentially labeled peptide will appear as a single MS peak, with differential abundances quantified during the MS/MS stage. Each of the above methods requires proteolysis of proteins, MS-based identification of each peptide, and quantitation analysis.

Quantitative methods for analysis of post-translational modifications are also being developed for both gel-based and LC-MS/MS proteomics methods. One of the most common forms of posttranslational modifications is phosphorylation, which is a key regulator in numerous functions including signal transduction, metabolic control and gene regulation. A quantitative analysis of phosphorylation patterns in the yeast pheromone pathway has been conducted (Gruhler et al. 2005), in which SILAC labeling was used in combination with phosphopeptide enrichment and three-stage MS. Over 700 identified phosphopeptides were identified and 139 of these were differentially regulated in response to the pheromone alpha-factor, including a number of cell-cycle proteins. Phosphopeptides were specifically targeted in the third stage MS by selection of peaks with a neutral loss corresponding to the phosphate group, increasing the specificity and improving detection of post-translational modification. Combinations of techniques such as these can permit targeting of specific low abundance proteins and/or post-translational modifications, and will greatly aid in pathway elucidation.

Looking beyond the gel or MS based proteomics tools for protein quantitation, applications of protein arrays represent a relatively new technology that is being developed to generate high-throughput data on proteins and protein interactions (see Cretich et al. 2006; Stoll et al. 2005 for reviews). The most popular approaches to date have been the use of a variety of monoclonal or polyclonal antibodies reactive to a subset of protein targets (e.g. phospho-proteins, Gembitsky et al. 2004), and the development of microarrays for protein expression (Kopf et al. 2005; Wingren and Borreback, 2004). Protein microarray methods are reminiscent of genome-wide DNA microarray technologies that have been providing powerful and informative quantitation of gene expression networks. Once established, protein microarray technology should provide an effective means of rapidly quantifying protein expression at the proteome level.

Many of these methods have their own limitations in providing quantitative data for systems biology analysis. Indeed, recently acquired systematic quantitation data have come more from classical methodologies such as immunoprecipitation and western blotting rather than novel proteomics approaches, largely because these methods are simple and provide more accuracy in small-scale experimentation for specific sets of proteins. This reflects the importance of key unsolved issues facing quantitative proteomics: (1) isolation of functional proteins and complexes of proteins among the whole protein population; (2) sensitive detection of proteins in multiplexed samples; and (3) sufficiently high throughput data acquisition. However, once these challenges are surmounted proteomics techniques should provide accurate and detailed quantitative data suitable for constructing detailed models of the cell cycle and other cellular processes. Although proteomics approaches can pose significant technical challenges and have not been widely utilized in modeling of cellular systems to date, they hold a great deal of promise in modeling as they provide a means to directly monitor proteins and protein isoforms within networks.

## Integration of Systems-Level Experimental Data

Given the numerous advantages of working with *S. cerevisiae*, it is not surprising that this organism has been at the forefront of efforts to quantitatively model a variety of cellular processes. In one of the first direct attempts at systems-level modeling, Ideker et al. (2001) applied systematic perturbations to galactose pathway components in yeast, and used microarray gene expression, quantitative proteomics, and physical interaction data to monitor pathway responses. From these data, a model of galactose pathway regulation in yeast was developed. In this paper, a general approach to systems level modeling was proposed, consisting of (i) pathway component definition, (ii) systematic perturbation of components, (iii) integration of data into the proposed model, and (iv) hypothesis generation based on deviations from the model. These steps are repeated, leading to successive model refinements.

In addition to the application of systematic perturbations, inherently cyclic processes such as the cell cycle may also be monitored over time. Recently, the first global proteome analysis of the cell cycle in *S. cerevisiae* was achieved using high-throughput MS/MS data and ICAT labels (Flory et al. 2006). In this study, synchronized cultures of yeast were sampled over the course of the cell cycle, and expression of 48% of open reading frames were quantified. Interestingly, no significant correlation was found with gene expression levels determined from previous microarray studies. Although integration of the data into a model of the cell cycle was not attempted, this study could provide valuable data for comprehensive integration into quantitative cell cycle models. More sophisticated computational tools for analyzing and comparing proteome-level experiments are also being developed (e.g. Prakash et al. 2006), which may lead to greater accuracy and coverage of expressed proteins.

Modeling efforts have also centered on characterizing the budding yeast pheromone response. In one case, the focus was on the G protein signaling involved in this process (Yi et al. 2003). Strains expressing fluorescently tagged versions of two G protein subunits, Gα (tagged with CFP) and Gγ (tagged with YFP) were constructed. A central feature of this response is the dissociation of Gα from both Gγ and a third G protein subunit, Gβ, in response to mating pheromone. By using fluorescence resonance energy transfer (FRET) to measure the dissociation of Gα-CFP and Gγ-YFP in a variety of mutant strain backgrounds, quantitative data was generated which could then be used for mathematical modeling. A second report (Kofahl and Klipp, 2004) presented a more comprehensive modeling effort characterizing the overall dynamics of the yeast pheromone pathway, incorporating data derived from numerous previously published studies. Klipp and colleagues (2005) have similarly integrated a large amount of experimental data relating to budding yeast osmoregulation to model the overall dynamics of the response to osmotic shock. These efforts represent an important advance in our understanding of a number of cellular processes, and offer the capability of predicting the behavior of individual components in response to perturbations to the system. It is important, however, to recognize that networks of proteins governing specific processes do not function in isolation in living cells. Indeed, there is typically interplay between the regulatory components involved in biological pathways that are conventionally described as distinct. An important step in obtaining mathematical models that are truly representative of living systems is to determine and incorporate the crosstalk between processes, as has recently been done for the pheromone and starvation pathways in budding yeast (Schaber et al. 2006).

From the data on genes, proteins, and pairwise interactions obtained using methods such as synthetic lethal and tagging studies and yeast two-hybrid assays, a representation of the network of pathways and interactions occurring within the cell and, in particular, the sub-network responsible for regulation of the cell cycle can be constructed. In addition to this network architecture, one must also account for the time dependence of the system and its response to perturbations in order to gain an understanding of overall cellular behavior. These factors may be addressed through dynamic modeling.

## Analysis and Interpretation through Dynamic Modeling

Given the complexity of many of the underlying networks it is essential to provide a mathematical framework to augment our intuitive understanding of their behavior. In this section we outline the progress made in applying mathematical analyses to investigations of the cell division cycle, much of which has focused on budding yeast. The first ordinary differential equation (ODE) based models of the eukaryotic cell cycle were proposed in the mid 1970’s (cf. chapter 10 of Goldbeter, 1996, for a comprehensive review of early activity in this area). These early models were constructed in the absence of biochemical detail; the proposed oscillatory mechanisms were inspired by chemical networks with periodic behavior (e.g. Kauffman and Wille, 1975). Once molecular details of cell cycle regulation began to emerge, a new generation of models emerged in an attempt to more accurately describe the process (Hyver and Le Guyader, 1990; Goldbeter, 1991; Tyson, 1991; Norel and Agur, 1991). These models address cell cycle progression in amphibian embryonic cells, during the stage in which nuclear division is decoupled from cell growth. In this case the key step in cell cycle progression is entry into mitosis, driven by the Maturation Promoting Factor, MPF, a heterodimer composed of cyclin and a cyclin-dependent kinase. These models were constructed in an attempt to understand how the interaction between these two proteins could lead to oscillatory behavior.

Since then, much effort has been dedicated to incorporating our increasing understanding of the molecular basis for cell cycle progression into models that can account for the system’s behavior. These attempts to elucidate the general principles underlying the eukaryotic cell cycle have been reviewed in Ingolia and Murray (2004). Recent contributions include Csikász-Nagy et al. (2006), Srividhya and Gopinathan (2006) and Yang et al. (2006), the latter of which describes a spatiotemporal model. The data used to inform these models is collected from specific organisms, each of which employs unique elements in cell cycle regulation. Consequently, an additional complement to the investigation of the generic mechanism of the cell cycle is the description of the specific aspects found in particular species. Recent examples of these species-specific modeling efforts include *Xenopus* (Sha et al. 2003), fission yeast (Novak and Pataki, 2001; Sveiczer et al. 2001), and mammalian cells (Chassagnole et al. 2006: Novak and Tyson, 2004; Qu et al. 2003; Sawt et al. 2004). The most extensive modeling efforts however have been made in the context of budding yeast, to which we now turn.

As outlined above, the experimental community has had great success in uncovering the molecular details of budding yeast physiology. As such, there is a wealth of data available for the construction of mathematical models of the yeast cell cycle. A number of distinct approaches have been taken in an attempt to reconcile the known biochemical interactions with the observed dynamic phenomena.

Li et al. (2004) describes a simple Boolean model of the cell cycle process. The authors propose a model composed of 11 proteins, including Clb5 and Clb6, the cyclins which bind to the cyclin-dependent kinase Cdc28 to form the budding yeast Maturation Promoting Factor. The interactions contained in the model were extracted from the literature; no parameter fitting was performed. In standard Boolean fashion, this model describes lockstep progression of the proteins between their active and inactive forms. The analysis stresses the stability of normal cell-cycle behavior among all possible model configurations, and the authors suggest that this may be an illustration of the general level of stability of existing biochemical networks. A similar conclusion is reached in Wang et al. (2006) and Wang and Han (2007), in which the authors investigate the energy landscape of a simple stochastic model of the yeast cell cycle.

An alternative approach is taken in Chen, H.-C. et al. (2004), in which the authors present a systematic model-construction algorithm driven by gene expression data. The algorithm is illustrated by application to microarray data on genes implicated in the budding yeast cell cycle. The resulting model represents a characterization of the transcriptional regulatory network underlying the cell cycle process. This is a promising approach for hypothesis-generation based on time-series microarray data. However, as the authors point out, its use in this case suffers from the limitation that expression data can only indirectly reveal posttranslational regulation and other modifications known to be integral to cell cycle progression.

The papers discussed above use the budding yeast cell cycle as an illustration of more general analytic issues. In contrast, the model presented by Chen et al. (2000) has as its main purpose the elucidation of the budding yeast cell cycle. This model involves nine independent protein concentrations whose time-evolution is governed by a set of nonlinear ODE’s involving about 50 parameters. The interactions were identified from the literature, and the specific form of the reaction rates were based on standard biochemical kinetics. Some parameter values are provided by experimental data on kinetics, but many are fit manually to training data. This data consists of descriptions of genetic perturbations (knockouts and altered gene dosage) on the length of G_1_ phase and the cell mass at various points in the cycle. The model succeeds in accounting for a great deal of molecular detail on cell cycle regulation. It is able to predict the result of a number of genetic perturbations, and so can be used to probe hypotheses about novel experiments or mechanisms.

Cross et al. (2002) present experimental results designed as a systematic test of the validity of the model in Chen et al. (2000). Using both morphological and molecular observations, Cross et al. were able to identify aspects of the model which provide robust predictions (e.g. dependence of cell size on CLN3 dosage) and areas in which improvement is required (e.g. interactions between G_1_ cyclins and Cdh1). Overall, the experimental results confirm that the model of Chen et al. (2000) is a worthy attempt at describing the dynamic complexities of the budding yeast cell cycle.

Validation of the sort presented in Cross et al. (2002) is an essential component of the analysis and evolution of mathematical models. In the absence of interplay with experiments, modeling quickly becomes a sterile activity. Conversely, the activity surrounding the Chen model is an example of the opportunities that such models provide for improving our understanding of complex phenomena. For example, the model has been used to investigate the underlying mechanisms of the cell cycle both experimentally (Cross, 2003) and theoretically (Battogtokh and Tyson, 2004). Moreover, extensions to the model have been proposed: a complementary model of the morphogenesis checkpoint appeared Ciliberto et al. (2003), and the model was used to provide the “background” for a model of cell growth and the G_1_/S transition in Alarcón and Tindall (2007).

An extended version of the Chen model appeared in Chen, K. C. et al. (2004), in which details of the M-G_1_ transition are incorporated into the model. This paper also describes an extensive validation against more than 100 mutant phenotypes, the vast majority of which provide agreement with model predictions.

The model in Chen, K. C. et al. (2004) could be described as the state-of-the-art in terms of dynamic description of cell cycle regulation in budding yeast, but it is hardly the end of the story. Any model represents a working hypothesis, and can be improved by refinement or extension. Extensions of this model will occur in a number of directions. The current description necessarily provides only a very abstract representation of certain aspects of the cell cycle machinery. These include spindle and bud formation, the mechanism by which cell size is sensed (as addressed in Alarcón and Tindall, 2007, and Barberis et al. 2007), the initiation of DNA replication, and the DNA replication process itself. In each such case, it may be that the mechanism under consideration can be given a dynamic description that is relatively modular, in the sense that interactions within the module are more dense than interactions between modules. Our group is currently undertaking the construction of a dynamic model of the initiation of DNA replication. This mechanism involves a number of interacting molecular factors, but there are relatively few interactions between these initiation factors and the other components of the Chen, K. C. et al. (2004) model. Whether such modularity is an inherent biological design principle or simply a byproduct of biases in our analysis techniques is currently a topic of debate (Lauffenburger, 2000; Wolf and Arkin, 2003; Szallasi, Periwal and Stelling, 2006).

Refinements of this type of model may come in the form of corrections to the underlying interaction map, the presumed form of the reaction kinetics, or the model parameters (primarily kinetic constants). The topology of the interaction map is the most robust information on which the model is based. It is typically confirmed by a number of previously reported experimental results and so should only be altered if those experimental findings are drawn into question. The individual interactions are described by standard functional forms (e.g. mass action, Michaelis-Menten, Goldbeter-Koshland switch). The decision as to which form to use is based on knowledge of the underlying chemical mechanism when it is available. Otherwise, intuition is followed, along with the guiding principle of Occam’s Razor that simple descriptions should be presumed in the absence of any evidence to the contrary. This *ad hoc* procedure leaves room for improvement as more details of molecular events are revealed. An alternative approach is to use an algorithm to “fit” the functional form for the kinetic events to experimental results, as presented in Sugimoto et al. (2005). This “bottom up” approach can be lauded for the primary role that experimental findings play in dictating the form of the model, but an ideal approach would also take advantage of known details of chemical kinetics.

The most subtle form of model refinement is improvement in the choice of parameter values representing kinetic constants. In some cases these have been measured directly (e.g. by enzymological assays) or can be inferred (e.g. degradation rates from *in vivo* half lives). However, in a complex model such as that described by Chen, K. C. et al. (2004), the majority of these parameter values are indirectly represented in the behavior of the system as a whole. The so-called “inverse problem” of choosing parameter values so that the model fits the experimental data is challenging, and a number of methods have been applied. A direct approach is to pose and solve an optimization problem to minimize the difference between the model’s prediction and the experimental findings, as carried out in Moles et al. (2003), Rodriguez-Fernandez et al. (2006), and Tsai and Wang (2005), for example. While this method is often successful, its utility can be argued in the case of a model of biochemical events, where one frequently has a lack of confidence in the model formulation and/or the experimental measurements. In cases such as these, some groups have argued that rather than identifying a single “best fit” parameter set, it is more useful to report the region in parameter space over which the model is a valid description of reality. This approach was taken in Battogtokh et al. (2002), in which a best fit probability distribution was given for a model of a genetic regulatory circuit. This method was expanded on in Brown and Sethna (2003) and in Brown et al. (2004) in which the resulting probability distribution is made use of for model analysis.

In each case, the models described above attempt to provide a dynamic description of the abundance (and corresponding activity) of the proteins and protein isoforms that regulate cell cycle progression. Most of these efforts have relied on phenomenological or genetic data, and so could be verified only through these indirect measurements. The proteomics methods outlined in the previous section hold the promise of providing quantitative data on protein abundance which would allow direct comparison with model simulation, and so will provide more immediate and higher-confidence model fidelity.

## Future Directions

In order to truly represent the complexity of the cell cycle, future efforts will need to incorporate a number of aspects of cellular growth and division that have been largely absent from the models established to date. A major challenge is to reflect the myriad roles played by various types of RNA. Although assessment of genome-wide mRNA levels has now become a standard practice, pools of tRNA and rRNA that regulate translation, as well as microRNAs and their effect on gene expression (Kloosterman and Plasterk, 2006) may also have to incorporated. Further consideration of changes in both the inter- and extracellular environments, including pH, temperature, ion concentrations, and nutrient availability are all elements potentially required within realistic models. Additionally, as mentioned above, the extent of crosstalk between networks of factors governing different processes will have to be determined.

There remain some technical challenges in data collection for precise computational modeling of the cell cycle. The majority of cell cycle proteins are low abundance proteins, limiting their detection and accurate quantitation using most established methods. Typically, cell cycle proteins are extracted from a large number of synchronized cells, so data is by necessity integrated, averaging out individual variation. It is also difficult to identify transient interactions occurring within a short time scale. Interactions of this type are not readily assessed using standard high throughput tools, but can often be addressed experimentally on a case-by-case basis. Molecular modeling may also provide insight into this problem through effective prediction of protein-protein structural interactions, though this is not readily achieved using existing computational tools.

Future challenges to the modeling framework will involve the extension of existing models across spatial and temporal scales. In part, this will require the use of complementary methodologies (e.g. stochastic methods for addressing networks with low copy number, partial differential equations to address issues of cellular localization). Experimentally, spatial data may be collected via fluorescent labeling of targeted proteins. Here, tagged target proteins can be imaged and sub-cellular locations determined within individual cells, and the resulting spatial data incorporated into dynamic models. These cellular aspects must also be integrated into models of cancer progression addressing issues such as tumor growth (Byrne et al. 2006), angiogenesis (Chaplain et al. 2006) and response to therapy (Sachs et al. 2001). The work of Alarcón et al. (2004) represents a step in this direction.

Finally, the extent to which successful approaches to cell cycle modeling in yeast are adaptable to human cells will need to be assessed. Although a large proportion of yeast cell cycle factors have human orthologs that function in much the same manner, there are differences that will need to be accounted for, including the multiple CDKs present in human cells compared to a single one (Cdc28) in budding yeast, and factors such as p53 and geminin which are present in humans but not yeast. Researchers working with *S. cerevisiae* have long enjoyed the advantage of being able to easily knock-out or down-regulate specific protein factors, facilitating the ability to make targeted perturbations that can test the predictive ability of mathematical models. With the advent of siRNA knockdown technology, similar assessments of robustness can be conducted for models developed for human cells. Ultimately, one hopes that these efforts will result in a much clearer understanding of the molecular differences between normal and cancerous cells along with the development of drug targeting strategies that are effective at combating cancer with minimal side effects.

## Figures and Tables

**Table 1:** Online resources for yeast interactions, complexes and pathways.

**Database or resource name**	**URL link**	**Service and content**
***Yeast-specific resources:***		
Protein-Protein Interaction and Complex Viewer (MIPS-CYDB)	http://Mips.gsf.de/proj/yeast/CYGD/interaction/	Protein-protein interaction from large scale screenings
General Repository for Interaction Datasets (GRID)	http://Biodata.mshri.on.ca/grid	Central repository for yeast protein interactions
Curagene Drosophila/yeast interaction database	http://Portal.curagen.com/cgi-bin/interaction/flyHome.pl	Protein interactions
	http://Portal.curagen.com/cgi-bin/interaction/yeastHome.pl	
Yeast Protein Complex Database	http://Yeast.cellzome.com	Systematic analysis of multiprotein complexes in yeast
Saccharomyces Genome Database (SGD)	http://www.yeastgenome.org	Information of the broadest range on yeast biology, and inter-connected service with other public databases like YPD, GenBank, Medline, MIPS, SWISS-PROT, etc.
Prophecy Database	http://prophecy.lundberg.gu.se	Phenotypic collection for deletion strains of yeast
MIPS Comprehensive Yeast Genome Database (CYGD)	http://mips.gsf.de/genre/proj/yeast	Information on the molecular structure and functional network of annotated proteins in yeast
Yeast GFP Fusion Localization Database	http://yeastgfp.ucsf.edu	Protein localization data set using GFP fusion proteins in yeast
Yeast Proteome Database (YPD)	http://www.biobase-international.com	Annotation focuses on the molecular function and biological role of proteins, consequences of gene mutation, and the physical and regulatory interactions between proteins and genes
Invitrogen GFP Clone Collection	http://clones.invitrogen.com/cloneinfo.php?clone=yeastgfp	A collection of GFP-tagged yeast strain is available from Invitrogen, the database covers three-quarters of the yeast proteome and over two-thirds of previously unlocalized proteins.
Open Biosystem Yeast Collections	http://www.openbiosystems.com/Genomics	Yeast collections including ORF collection, Tet-Promoter Hughes collection, Yeast Knock Out collection, TAP tagged collection, HA tagged collection
European Saccharomyces Cerevisiae Archive for Functional Analysis (EUROSCARF)	http://web.uni-frankfurt.de/fb15/mikro/euroscarf/	Collections of systematic deletion mutants, TAP fusion strains, and degron strains
***General resources:***		
Database of Interacting Proteins (DIP)	http://Dip.doe-mbi-ucla.edu	Experimentally determined protein-protein interactions
Database of ligand receptor partners (DLRP)	http://Dip.doe-mbi.ucla.edu/dip/DLRP.cgi	Ligand-receptor complexes involved in signal transduction
Biomolecular Interaction Network Database (BIND)	http://www.blueprint.org/bind/bind.php	Molecular interactions, complexes and pathways
Molecular Interaction Database (MINT)	http://Cbm/bio.uniroma2.it/mint/	Protein interactions with proteins, nucleic acids and small molecules
Peptide Atlas	http://www.peptideatlas.org	Compendium of peptides identified by tandem mass spectrometry
Cytoscape	http://cytoscape.org	Visualization of interactions and integration of expression and state data
BioNetBuilder	http://err.bio.nyu.edu/cytoscape/bionetbuilder/	Cytoscape plug-in for creation of biological networks from multiple database sources
SBEAMS	http://www.sbeams.org/	Systems Biology Experiment Analysis Management System, a framework for managing systems level data
